# Expression and Characterization of Carotenoid Cleavage Oxygenases From *Herbaspirillum seropedicae* and *Rhodobacteraceae bacterium* Capable of Biotransforming Isoeugenol and 4-Vinylguaiacol to Vanillin

**DOI:** 10.3389/fmicb.2019.01869

**Published:** 2019-08-13

**Authors:** Zichun Han, Liangkun Long, Shaojun Ding

**Affiliations:** The Co-Innovation Center of Efficient Processing and Utilization of Forest Resources, Jiangsu Key Lab for the Chemistry & Utilization of Agricultural and Forest Biomass, College of Chemical Engineering, Nanjing Forestry University, Nanjing, China

**Keywords:** *Herbaspirillum seropedicae*, *Rhodobacteraceae bacterium*, vanillin, 4-vinylguaiacol, isoeugenol, carotenoid cleavage oxygenase

## Abstract

HsCCO and RbCCO from *Herbaspirillum seropedicae* and *Rhodobacteraceae bacterium* were selected and characterized from five putative bacterial carotenoid cleavage oxygenase gene sequences, due to merits in expression solubility and catalytic properties. Both enzymes can convert 4-vinylguaiacol and isoeugenol to vanillin. HsCCO showed maximum activity at 40°C and pH 7.0 and was stable at pH 6.5–10 and temperature around 25°C, retaining over 90 and 80% of initial activity, respectively. RbCCO showed maximum activity at 35°C and pH 9.0 and was stable at pH 6–11 and temperatures of 25–30°C, retaining over 80% of initial activity. The kinetic constants *K*_*m*_ of HsCCO for isoeugenol and 4-vinylguaiacol were 1.55 and 1.65 mM and *V*_*max*_ were 74.09 and 27.91 nmol min^–1^ mg^–1^, respectively. The kinetic constants *K*_*m*_ of RbCCO for isoeugenol and 4-vinylguaiacol were 2.24 and 0.85 mM and *V*_*max*_ were 76.48 and 19.96 nmol min^–1^ mg^–1^, respectively. The transformed *Escherichia coli* cells harboring *HsCCO* converted isoeugenol and 4-vinylguaiacol at molar conversion yields of 80 and 55% and the maximum vanillin concentrations were up to 1.22 and 0.84 g L^–1^, respectively. Comparably, the molar conversion yields of the transformed *E. coli* cells harboring *RbCCO* against isoeugenol 4-vinylguaiacol were 75 and 58%, and the maximum vanillin yields were up to 1.14 and 0.88 g L^–1^, respectively.

## Introduction

Vanillin (4-hydroxy-3-methoxybenzaldehyde) is one of the most important flavor additives worldwide. It is widely used in the food, pharmaceutical and the chemical industry. Natural vanillin is mainly derived from vanilla cells. Because the content of vanillin is low in vanilla cells and the cost of separation and extraction of vanillin is relatively high, natural vanillin accounts for only 1% of global production (Priefert et al., 2001; Schwab et al., 2018). Therefore, chemical vanillin has become the first choice for industrial applications due to its low price. However, the chemical synthesis of vanillin causes environmental pollution issues during the production process and chemically produced vanillin has adverse health effects (Goutam and Pritam, 2018).

The development of biotechnology has added more possibilities to the synthesis of vanillin (Mathew and Abraham, 2006; Kaur and Chakraborty, 2013). At present, the main material source for the bioproduction of vanillin is usually similar to the structure of vanillin, such as eugenol, isoeugenol, and ferulic acid. Among them, ferulic acid is widely distributed in the whole plant community, and mainly exists as a component of the plant cell wall (Bento-Silva et al., 2018). The conversion of ferulic acid to vanillin by microorganisms has a long history of research. As early as 1996, Lesage-Meessenhad used two filamentous fungi to synthesize vanillin from ferulic acid. In the first step, *Aspergillus niger* transformed ferulic acid to vanillic acid and in the second step vanillic acid was reduced to vanillin by *Pycnoporus cinnabarinus*. However, the catalysis efficiency in the second step is low in specificity; the final yield is only 22% (Lesagemeessen et al., 1996). In addition to fungi, various bacterial species such as *Streptomyces setonii* ATCC 39116 (Fleige et al., 2013), *Bacillus subtilis* B7-S (Chen et al., 2016), and *Pseudomonas putida* KT2440 (Plaggenborg et al., 2003) have been reported to produce vanillin from ferulic acid.

Several metabolism pathways from FA to vanillin have been identified in bacteria (Priefert et al., 2001). Among them, a high conversion can be obtained by wild-type or engineered bacterial cells using the deacetylation of the coenzyme-dependent deacetylation pathway (Gallage and Møller, 2015; Goutam and Pritam, 2018; Khoyrattya et al., 2018), which involves the activity of feruloyl-CoA synthetase (Fcs) and enoyl-CoA hydratase/aldolase (Ech) enzymes. For example, Chakraborty et al. (2016) used recombinant *Escherichia coli* (fcs^+^/ech^+^) to transform ferulic acid and finally obtained 68 mg/L of vanillin. They also used recombinant *Pediococcus acidilactici* BD16 carrying Fcs and enoyl-CoA hydratase genes to transform ferulic acid and finally obtained 4.01 g/L of vanillin (Chakraborty et al., 2017). However, since Fcs require CoA and ATP as coenzymes, this increases the complexity and cost of the system.

Isoeugenol is an aromatic compound similar in structure to ferulic acid, usually derived from essential oils. Isoeugenol is an important precursor for the industrial synthesis of vanillin (Priefert et al., 2001; Zhao et al., 2018). It was reported in 1999 that *Bacillus* sp. produced 0.61 g/L vanillin (molar yield 12.4%) with isoeugenol as the sole carbon source (Shimoni et al., 2000). So far, it was found that various bacterial strains, such as, *P. nitroreducens* jin1 (Ryu et al., 2013), *P. putida* IE27 (Yamada et al., 2007a), *B. subtilis* (Zhang et al., 2006), and *Trichosporon asahii* (Ashengroph and Amini, 2017), etc., can convert isoeugenol to vanillin. The highest molar conversion yield can reach about 80%, but some reactions were accompanied by the production of vanillic acid (Zhang et al., 2006; Ashengroph and Amini, 2017). Isoeugenol monooxygenase was considered as the enzyme responsible for the biotransformation of isoeugenol to vanillin in these species (Yamada et al., 2007b, 2008; Ryu et al., 2013). This enzyme has been found to be highly homologous with carotenoid cleavage oxygenase family proteins based on the sequence alignment. Carotenoid cleavage oxygenases catalyze the oxidative cleavage of conjugated C=C bonds of organic molecules without the need for any coenzyme (Marasco and Schmidt-Dannert, 2008). In plant, carotenoid cleavage oxygenases involve in the conversion of β-carotene to abscisic acid via the BCH (β-carotenehydroxylase) pathway (Espasandin et al., 2014).

Recently, Furuya et al. (2014) found that the oxygenase CsO2 belonging to carotenoid cleavage oxygenase family from *Caulobacter segnis* ATCC 21756, can catalyze both 4-vinylguaiacol and isoeugenol to vanillin. Vanillin was effectively produced from ferulic acid at an 80% of overall conversion yield by using *E. coli* cells harboring the decarboxylase/oxygenase cascade, in which FA was first decarboxylated to 4-vinylguaiacol by phenolic acid decarboxylase and then oxidized to vanillin *via* oxygenase (Cso2) (Furuya et al., 2014, 2015). Coenzyme is not required in these reactions, which reduces the complexity and cost of the reaction. The decarboxylase has been well studied and the bioconversion of FA to 4-vinylguaiacol by phenolic acid decarboxylase is very efficient (Furuya et al., 2015; Hu et al., 2015; Chen et al., 2018). However, there is less understanding of oxygenases responsible for the conversion of 4-vinylguaiacol to vanillin compared with decarboxylases. Its low thermal stability, insoluble expression and low catalytic activity severely restrict its industrial application. So far, only a few oxygenases have been identified from bacteria.

In fact, many bacterial species in nature such as *B. coagulans* (Karmakar et al., 2000) and *Enterobacter* sp. *Px6-4* (Li et al., 2008) have been reported to convert ferulic acid to vanillin *via* 4-vinylguaiacol as an intermediate. Genome searching using CsO2 as the probe by BLASTP may find a number of homogenous sequences in various bacterial genomes. In order to comprehensively understand the function of these putative carotenoid oxygenase family proteins, five carotenoid oxygenase family protein sequences were selected from different bacterial species and the characters of corresponding proteins were compared in the aspects of their expression solubility in an *E. coli* BL21 (DE3) strain and catalytic properties. Finally, two novel proteins, HsCCO and RbCCO, derived from *Herbaspirillum seropedicae* and *Rhodobacteraceae bacterium*, respectively, were screened based on the biochemical properties and their potentials in converting 4-vinylguaiacol and isoeugenol to vanillin were further evaluated in this work.

## Materials and Methods

### Chemicals

Isoeugenol was purchased from the Macklin Group (Shanghai, China); 4-vinylguaiacol was obtained from the LID Group (Shijiazhuang, China); vanillin was provided from Sigma-Aldrich (St. Louis, MO, United States). Isoeugenol and 4-vinylguaiacol were dissolved in dimethyl sulfoxide as stock solutions of 500 mM. The dimethyl sulfoxide and other organic compounds were obtained from Sinopharm Group (Beijing, China). The standard protein marker for sodium dodecyl sulfate polyacrylamide gel electrophoresis (SDS-PAGE) was purchased from Takara (Dalian, China). The kits used for bacterial plasmid extraction was purchased from Transgene (Beijing, China). All other chemicals were of analytical grade.

### Cloning and Expression of Five Carotenoid Oxygenases

Five codon-optimized genes based on the sequences of putative carotenoid oxygenases from *H. seropedicae* (*HsCCO*, GenBank accession no. WP_069373448.1), from *R. bacterium* (*RbCCO*, GenBank accession no. OHC44943.1), from *Kaistia soli* (*KsCCO*, GenBank accession no. WP_073057496.1), from *Agrobacterium vitis* (*AvCCO*, GenBank accession no. WP_012654221.1), and from *Rhodococcus aetherivorans* (*RaCCO*, GenBank accession no. WP_029541323.1) were synthesized by the Springen Group (Nanjing, China). The DNA fragments of *AvCCO* and *RaCCO* were digested with *Eco*RI/*Xho*I and inserted into pET-28a (+) (Novagen, Copenhagen, Denmark). The DNA fragment of *RbCCO* was digested with *Bam*HI/*Xho*I and inserted into pET-29a (+) (Novagen). The DNA fragments of *HsCCO* and *KsCCO* were digested with *Nde*I/*Xho*I and inserted into pET-29a (+) (Novagen). *E. coli* Top 10 and BL21 (DE3) (TransGene, Beijing, China) were used as host strains for gene cloning and expressing, respectively. The recombinant *E. coli* cells were cultured in LB medium (50 ml in 250 ml flasks) supplemented with 50 mg/ml kanamycin with shaking (200 rpm) at 37°C. To improve the solubility of RbCCO, the pGro7 plasmid carrying chaperonin genes groEL and groES was simultaneously transformed into *E. coli.* For *E. coli* carrying pGro7, the medium was further supplemented with chloramphenicol (20 mg/ml) and L-arabinose (3 mg/ml). After culturing for 2–2.5 h (OD_600_ = 0.8–1.0), IPTG (0.2 mM) was added and culturing was continued for an additional 16 h at 25°C. For optimizing expression of HsCCO and RbCCO in *E. coli*, the effect of induction temperature (15–30°C), IPTG concentration (0.1–0.8 mM), time (6–16 h), Fe^2+^ (0 or 1 mM) on the enzyme expression was investigated step by step in shaking flasks. Cells were harvested by centrifugation (5000 × *g*, 10 min, 4°C) and disrupted through ultrasonication in lysis buffer (50 mM NaH_2_PO_4_ and 300 mM NaCl pH 8.0). The enzyme activity in crude induced cell extracts was determined under the standard assay condition.

### Purification of Recombinant CCOs

The induced cell pellets resuspended in lysis buffer were disrupted by ultrasonication at an ice bath. After centrifugation (10,000 × *g*, 4°C, 10 min), the supernatants were applied to a Ni-NTA agarose gel and recombinant proteins were purified according to the manufacturer’s instructions. The concentration of the purified recombinant enzymes was measured using a BCA protein assay kit (Sangon, Shanghai, China). The purified enzymes were stored at 4°C and used as soon as possible for the following studies. The purity and molecular weight of the purified enzymes were estimated by SDS-PAGE.

### Effects of pH, Temperature and Metal Ions on Enzyme Activity and Stability

Optimum pHs and temperatures were determined using isoeugenol and 4-vinylguaiacol as substrates. Optimal pHs were determined in buffers at a range of pH 5.0–12.0 [0.2 M sodium phosphate buffer (pH 5.0–8.0 and 10% glycerol) and 0.2 M NaOH-glycine buffer (pH 8.0–12.0, and 10% glycerol)]. Optimum temperatures were determined in a range of temperature from 20 to 45°C. Results are expressed as the percentage of enzyme activity measured at the optimal pH or temperature.

In order to evaluate the temperature stability, enzymes were preincubated at different temperatures and the residual enzyme activities were determined after a specific time. For pH stability, the enzymes were preincubated in a buffer of pH 5.0–11.0 at 4°C for 8 h, then the residual enzyme activities were evaluated under assay condition.

To investigate the effect of metal ions on enzyme activity, metal ions (final concentration 1 and 5 mM, respectively) were simultaneously added to the reaction system and the enzyme activity were assayed in the presence of individual metal ions. The reactions without adding any chemicals were used as a control.

### Time Course of Whole Cell Production of Vanillin

Before the time course of whole cell production of vanillin, the effect of temperature, pH, shaking speed, and substrate concentration on whole cell transformation efficiency was determined using the transformed *E. coli* cells carrying *HsCCO* and *RbCCO*, respectively. The transformed *E. coli* cells were induced as described above, harvested by centrifugation (8500 × *g*, 10 min, 4°C) and washed three times with sodium phosphate buffer (0.2 M, pH 7.0) or NaOH-glycine buffer (pH 9.0) containing glycerol (10% v/v), respectively. The whole cell catalysis was carried out in a 50 ml Erlenmeyer flask with a reaction volume of 1 ml. The reaction system contained 80 mg (wet weight) of whole cells, 10 mM substrate (for temperatures, pH and shaking speed optimization) and 0.2 M sodium phosphate buffer (pH 7.0) or NaOH-glycine buffer (pH 9.0) containing glycerol (10% v/v) for HsCCO and RbCCO, respectively, at different temperatures, pH, shaking speed and substrate concentrations. After the end of the reaction, 2 ml of methanol was added to inactivate the reaction. Product concentrations in reaction mixture were determined by HPLC. In order to evaluate the effect of the organic solvents on the whole cell catalysis efficiency, the whole cell catalysis was carried out under optimal conditions in the presence of a 10% (v/v) organic solvent for 24 h. The product was analyzed by HPLC as described as above. The reactions without an organic solvent were used as a control. Time course of whole cell production of vanillin was performed by using the transformed *E. coli* cells carrying *HsCCO* and *RbCCO*, respectively, at an optimum pH and temperature for 24 h. At the time indicated, three parallel samples were taken out and inactivated with 2 ml of methanol. The products were quantified using HPLC.

### Enzyme Activity Assay and Product Analysis

Enzyme activity is determined by HPLC (Agilent Technologies 1260 infinity, Agilent, Palo Alto, CA, United States) using isoeugenol and 4-vinylguaiacol as substrates as previously described (Tang et al., 2018). The reaction mixture (500 μl) contains 2 mM substrate, sodium phosphate buffer (0.2 M and 10% glycerol at pH 5.0–8.0) or NaOH-glycine buffer (0.2 M and 10% glycerol at pH 8.0–12.0) and the appropriate number of enzymes. The reactions were performed at optimal temperature with vigorous shaking for 1 h. One unit of enzyme activity (μmol/min) is defined as the amount of the enzyme that catalyzes the conversion of specific substrate to form one micromole of vanillin per minute under the specified conditions of the assay method. The formed product was analyzed using HPLC as described previously by Hu et al. (2015). Identification of the formed product was performed by gas chromatography–mass spectrometry (GC–MS) (Agilent 7000B) with a DB-5MS column (length, 30 m; diameter, 0.25 mm; Agilent). Electron impact (EI) ionization was performed at electron energy of 70 eV. The ion source temperature and the transfer line temperature were set to 250°C. The column oven temperature programs were set to 60°C for 2 min (4-vinylguaiacol as substrate) or 3 min (isoeugenol as substrate), then to 300°C for 12 min with 20°C/min. Carrier gas was helium at 1 ml/min. The components were identified based on the comparison of their relative retention times and mass spectra with those of the established standards (NIST05 library data of the GC–MS system).

## Results

### Expression and Purification of the Recombinant Five Oxygenases

**FIGURE 1:**
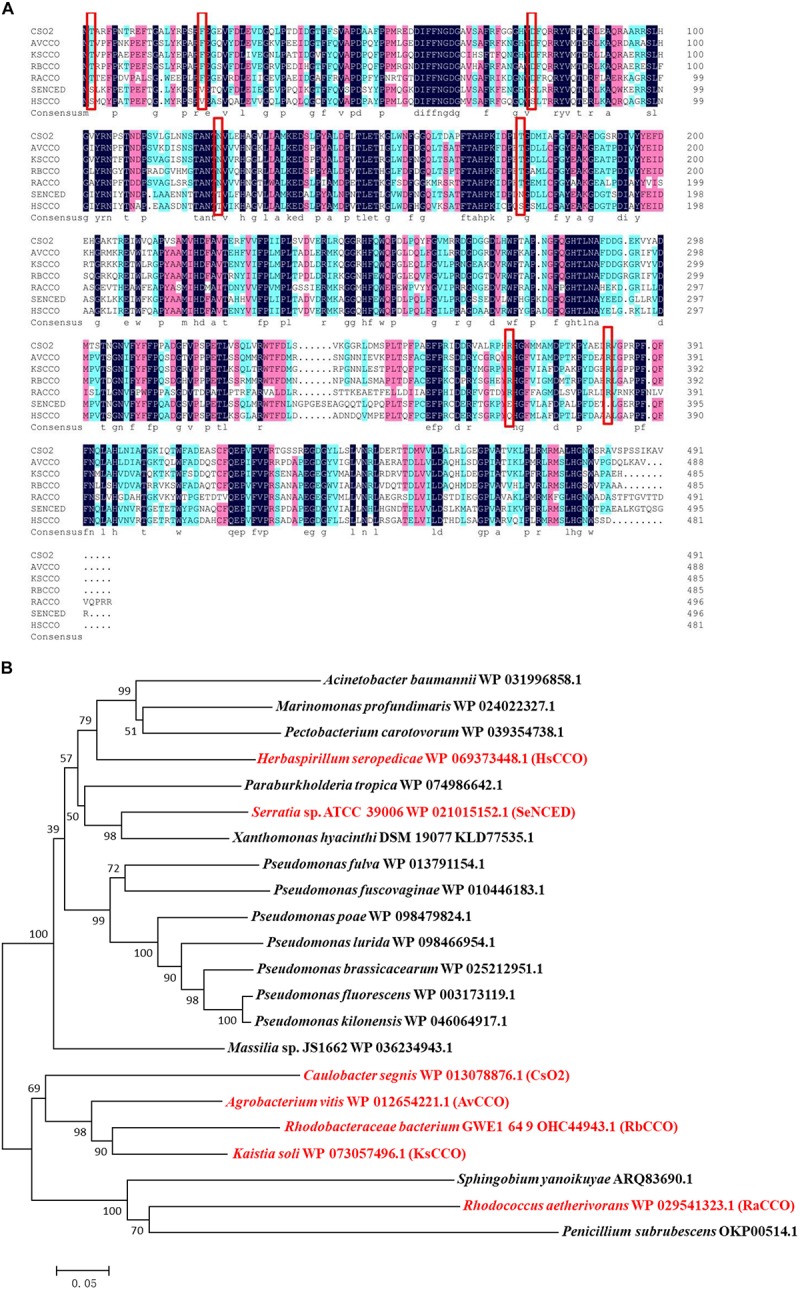
**(A)** Multiple alignments of seven amino acid sequences of the characterized CCOs from different bacteria in this work and in previous literature. Identical residues are black and conserved residues are other colors. Cso2 from *Caulobacter segnis* 21756 (WP_013079514.1), AvCCO from *Agrobacterium vitis* (WP_012654221.1), KsCCO from *Kaistia soli* (WP_073057496.1), RbCCO from *Rhodobacteraceae bacterium* (OHC44943.1), RaCCO from *Rhodococcus aetherivorans* (WP_029541323.1), SeNCED from *Serratia* sp. ATCC 39006 (WP_021015152.1), HsCCO from *Herbaspirillum seropedicae* (WP_069373448.1). **(B)** The phylogenetic trees resulting from analysis of carotenoid cleavage oxygenases of 22 amino acid sequences using Neighbor-Joining method. The characterized carotenoid cleavage oxygenases in this work and in previous literature are indicated with a red symbol and others with a black symbol. Numbers on nodes correspond to percentage bootstrap values for 1000 replicates. The enzyme names, organisms, and sequences are given in the Supplementary Table S1.

Search for bacterial genomic sequences by BLASTP may find over 100 sequences of carotenoid oxygenase family proteins similar to CsO2 (with over 57% identities). Five putative carotenoid oxygenase gene sequences (*HsCCO* from *H. seropedicae* WP_069373448.1, *RbCCO* from *R. bacterium* OHC44943.1, *KsCCO* from *K. soli* WP_073057496.1, *AvCCO* from *A. vitis* WP_012654221.1 and *RaCCO* from *R. aetherivorans* WP_029541323.1) were selected because of the relative high amino acid identity (50–70%) to CsO2 (Figure 1A). In a phylogenetic tree of carotenoid cleavage oxygenases, *HsCCO* and *SeNCED* are clustered in the up group of the evolutionary tree (Figure 1B), whereas, *AvCCO*, *RbCCO*, *KsCCO*, and *RaCCO* are clustered with CsO2 in the down group of the evolutionary tree (Figure 1B). SDS-PAGE analysis revealed that most of the proteins of KsCCO, RbCCO, AvCCO, and RaCCO appeared in the precipitate fraction (Supplementary Figures S1–S3), even if KsCCO, AvCCO, and RaCCO were also co-expressed with chaperones. Furthermore, preliminary enzyme activity measurement of all five purified carotenoid oxygenases also revealed that KsCCO, AvCCO, and RaCCO displayed much lower activity than HsCCO and RbCCO (Supplementary Table S2). So HsCCO and RbCCO were selected for further research in this work.

**FIGURE 2:**
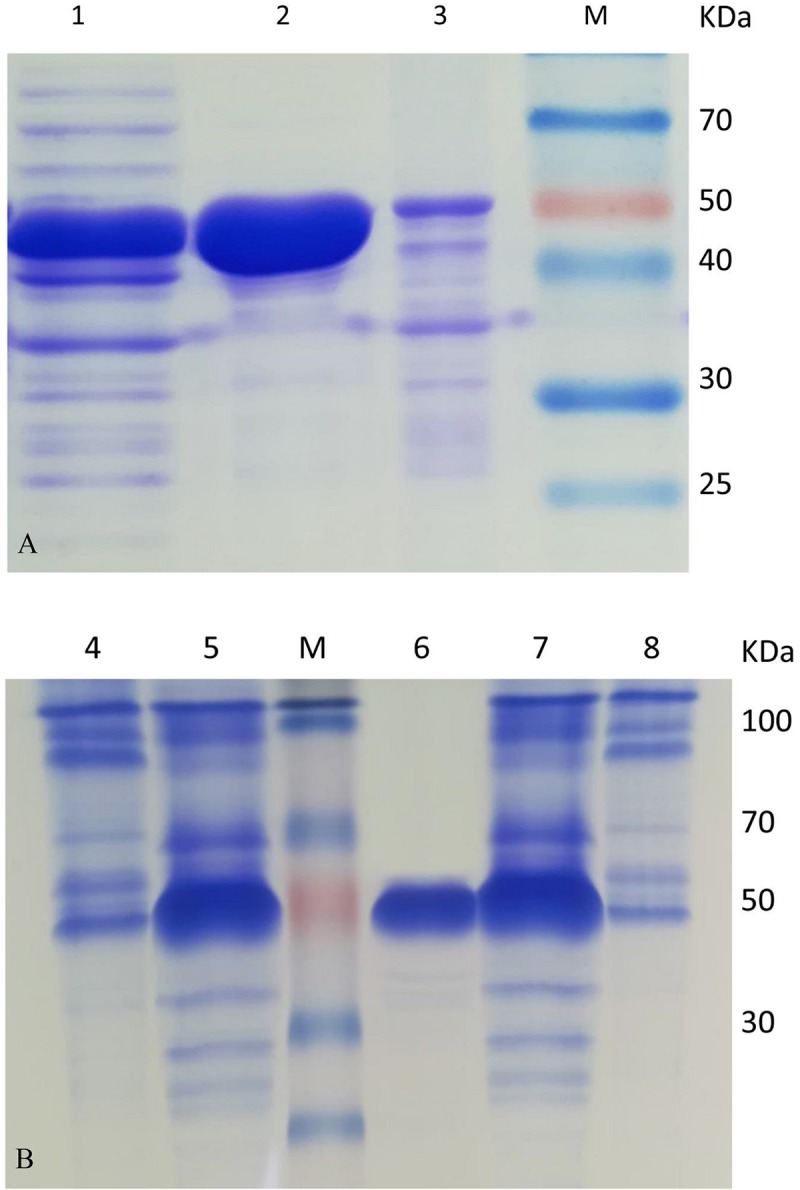
SDS-PAGE analysis of HsCCO **(A)** and RbCCO **(B)** solubility and purified protein. Lane 1–3: The supernatant fraction, purified HsCCO and precipitate fraction containing the target protein HsCCO. Lane M: Protein marker. Lane 4–5: The supernatant and precipitate fractions containing the target protein RbCCO without pGro7. Lane 6: The purified RbCCO. Lane 7–8: The supernatant fraction and precipitate fraction containing the target protein RbCCO with pGro7.

Most of the target protein of HsCCO appeared in the supernatant fraction, while the major target protein of RbCCO appeared in the precipitate fraction. To improve the solubility of RbCCO in *E. coli*, the gene *RbCCO* was co-expressed with plasmid pGro7 carrying chaperonin gene groEL and groES. As shown in Figure 2B, the insoluble protein in precipitate fraction was significantly reduced. The molecular masses of both purified proteins are about 55 KDa on SDS-PAGE, consistent with theoretic masses calculated from the amino acid sequences of the His-tagged proteins (Figure 2).

**FIGURE 3:**
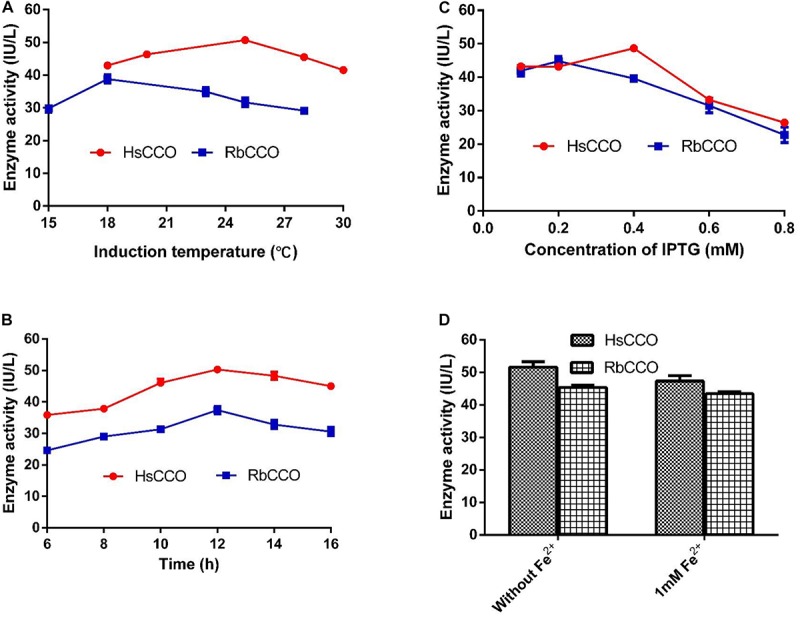
The optimization of culture conditions for HsCCO and RbCCO expression. **(A)** Induction temperature. **(B)** Induction time. **(C)** Concentration of IPTG. **(D)** Effect of Fe^2+^ on induction. The enzyme activity in crude cell extracts was determined under standard assay condition and defined as total unit per L of culture. Values shown are means of triplicate means + standard error (SE).

The culture conditions for HsCCO and RbCCO induction were optimized in shaking flasks. According to the experimental results, the optimal IPTG concentrations were 0.4 and 0.2 mM for HsCCO and RbCCO, respectively. The optimum induction temperatures were 25 and 18°C for HsCCO and RbCCO, respectively. According to literature reports, Fe^2+^ is used as a cofactor for carotenoid cleavage oxygenase (Kloer et al., 2005). However, the tested results indicated that no additional Fe^2+^ was required for HsCCO and RbCCO expression in this work (Figure 3).

### Effect of Temperature and pH on Enzymes Activity

**FIGURE 4:**
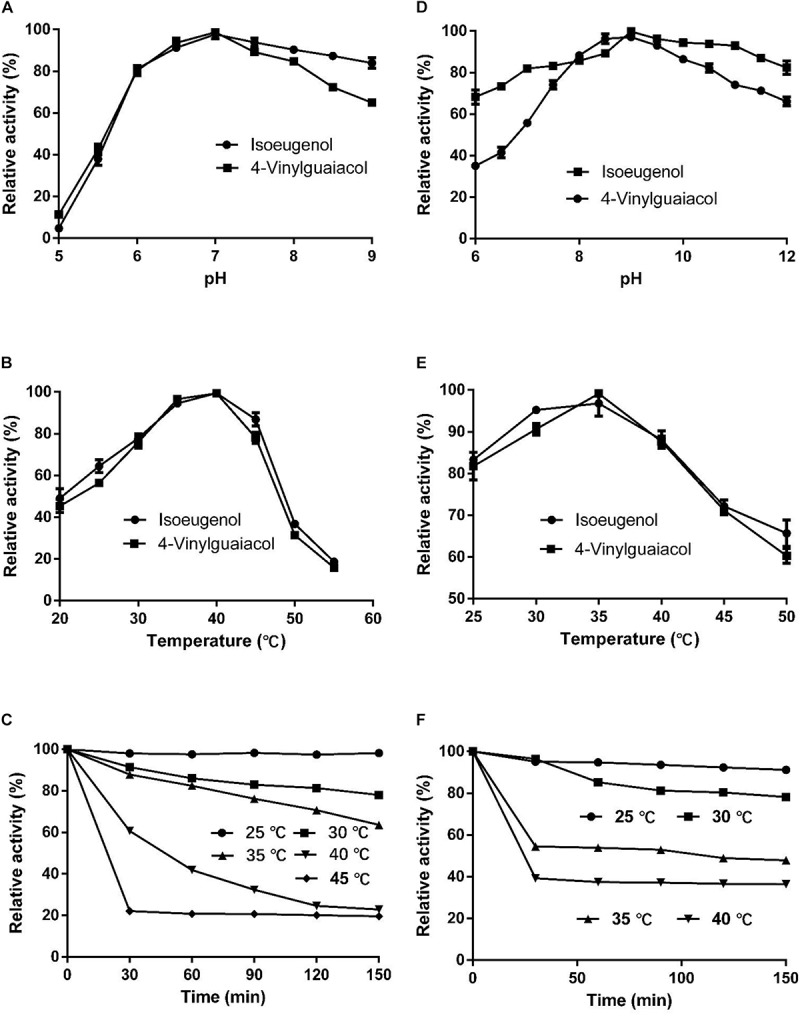
Effects of pH and temperature on enzyme activity and stability. **(A–C)** Effects of pH and temperature on HsCCO activity, and thermals stability, respectively. **(D–F)** Effects of pH and temperature on RbCCO activity, and thermals stability, respectively. Values shown are means of triplicate means + standard error (SE).

The optimal temperature for HsCCO with isoeugenol and 4-vinylguaiacol as substrate was 40°C and the optimal pH was 7.0 (Figures 4A,B). HsCCO was stable in the pH ranges of 6.5–10.0, maintaining more than 80% of its initial activity after incubation for 8 h (data not shown). HsCCO maintained nearly 100 and 80% activity after incubation at 25 and 30°C for 150 min, respectively, but seriously inactivated at temperature above 45°C (Figure 4C). The optimal temperature and pH of RbCCO were 35°C and 9.0, respectively (Figures 4D,E). RbCCO maintained more than 80% of its initial activity after incubation for 8 h in the range of pH 6.0–11.0 (data not shown). Similar as HsCCO, RbCCO maintained over 80% activity at 25–30°C, but 40% of its initial activity was still maintained at temperatures above 40°C (Figure 4F). Interestingly, the optimal pHs of AvCCO, RaCCO, and KsCCO ranged in alkaline from pH 9.0–10.0 (Supplementary Figure S4). The effect of metal ions on HsCCO and RbCCO activities were examined in the presence of several metal ions using isoeugenol as substrates (Supplementary Figure S5). The metal ions Co^2+^, Li^+^, Mg^2+^, and Zn^2+^ at 1 mM had almost no influence on the activity of HsCCO, but Li^+^ and Mg^2+^ at 5 mM slightly decreased HsCCO. Interesting, HsCCO enzyme activity was increased by 22% in the presence of 5 mM of Ca^2+^. For RbCCO activity, Co^2+^, Fe^3+^, and Li^+^ at 1 mM had no effect on the enzyme activity, but significant inhibitory effects was observed at 5 mM. The other tested metal ions had obviously inhibitory effects on RbCCO activity even at 1 mM.

### Kinetic Parameters of HsCCO and RbCCO

The kinetic parameters of HsCCO and RbCCO were measured under optimal conditions. With 4-vinylguaiacol as the substrate, *K*_*m*_ values were 1.65 and 0.85 mM, while *V*_*max*_ values were 27.91 and 19.96 nmol min^–1^ mg^–1^ for HsCCO and RbCCO, respectively. With isoeugenol as the substrate, *K*_*m*_ values were 1.55 and 2.24 mM, while *V*_*max*_ values were 74.09 and 76.48 nmol min^–1^ mg^–1^ for HsCCO and RbCCO, respectively. The results showed that the *V*_*max*_ of HsCCO and RbCCO against isoeugenol was much higher than that of 4-vinylguaiacol (Table 1), indicates that isoeugenol is more specific as a substrate than 4-vinylguaiacol. The transformed product vanillin from isoeugenol and 4-vinylguaiacol by the purified HsCCO and RbCCO were confirmed by GC-MS analysis (Supplementary Figures S6, S7).

**TABLE 1:** Kinetic parameters of HsCCO and RbCCO.

**Protein**	**Isoeugenol**		**4-Vinylguaiacol**	
	
	***K*_*m*_ (mM)**	***V*_*max*_ (nmol min^–1^ mg^–1^)**	***K*_*m*_ (mM)**	***V*_*max*_ (nmol min^–1^ mg^–1^)**
HsCCO	1.55	74.09	1.65	27.91
RbCCO	2.24	76.48	0.85	19.96

### Time Course of Whole Cell Production of Vanillin

**FIGURE 5:**
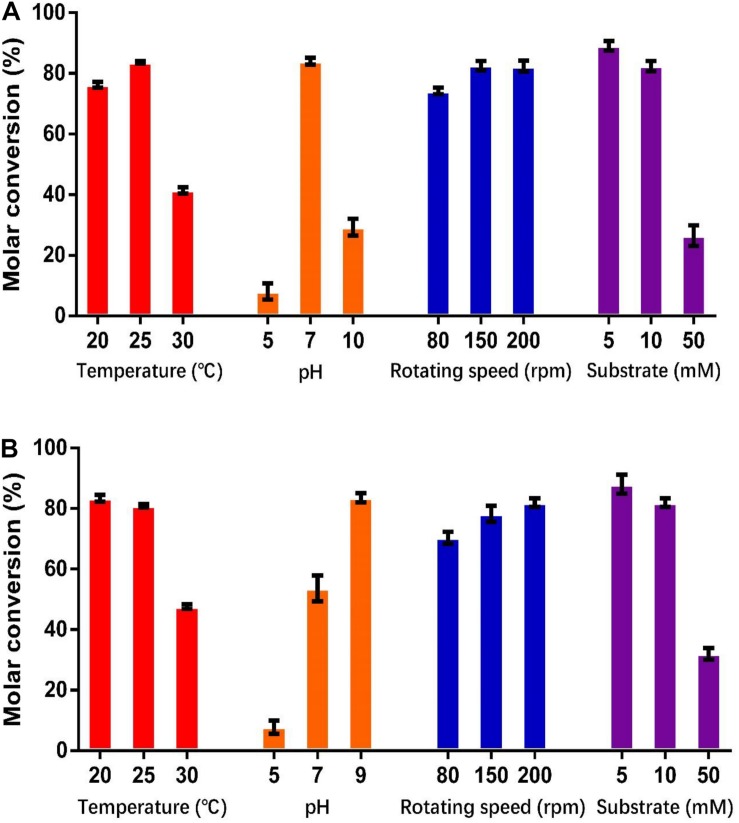
Optimization of temperature, pH, shaking speed, and substrate concentration for whole cell catalysis conditions for HsCCO **(A)** and RbCCO **(B)**. The molar conversion from 4-vinylguaiacol or isoeugenol to vanillin was calculated based on HPLC analysis. Values shown are means of triplicate means + standard error (SE).

As shown in Figure 5, the optimum catalysis temperatures for whole cells harboring genes *HsCCO* and *RbCCO* are 25 and 20°C, respectively, while the optimum catalysis pHs was 7 and 9, respectively, as same as the optimum pH of the free enzymes. Increasing the rotational speed benefited the whole cell conversion (Figure 5). The molar conversion yield was greatly reduced when the substrate concentration of isoeugenol and 4-vinylguaiacol was increased (Figure 5). Consequently, the following experiments were carried out in the best conditions.

**FIGURE 6:**
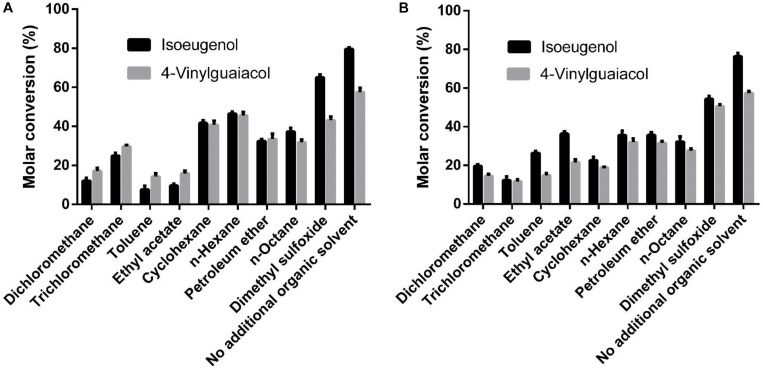
Effects of organic solvents on whole cell catalysis of HsCCO **(A)** and RbCCO **(B)**. The whole cell catalysis was carried out under the optimal conditions in the presence of 10% (v/v) different organic solvent for 24 h and the molar conversion from 4-vinylguaiacol or isoeugenol to vanillin was calculated based on HPLC analysis. Values shown are means of triplicate means + standard error (SE).

As shown in Figure 6, the whole cell conversion yield was obviously decreased when co-incubation with 10% additional organic solvent. Dimethyl sulfoxide has the least negative effect on the conversion yield in all organic solvents, but the HsCCO conversion yield was still decreased by 18% on isoeugenol and by 17% on 4-vinylguaiacol, and the RbCCO conversion yield was decreased by 29% on isoeugenol and by 12% on 4-vinylguaiacol compared to that of no additional organic solvent (Figure 6).

**FIGURE 7:**
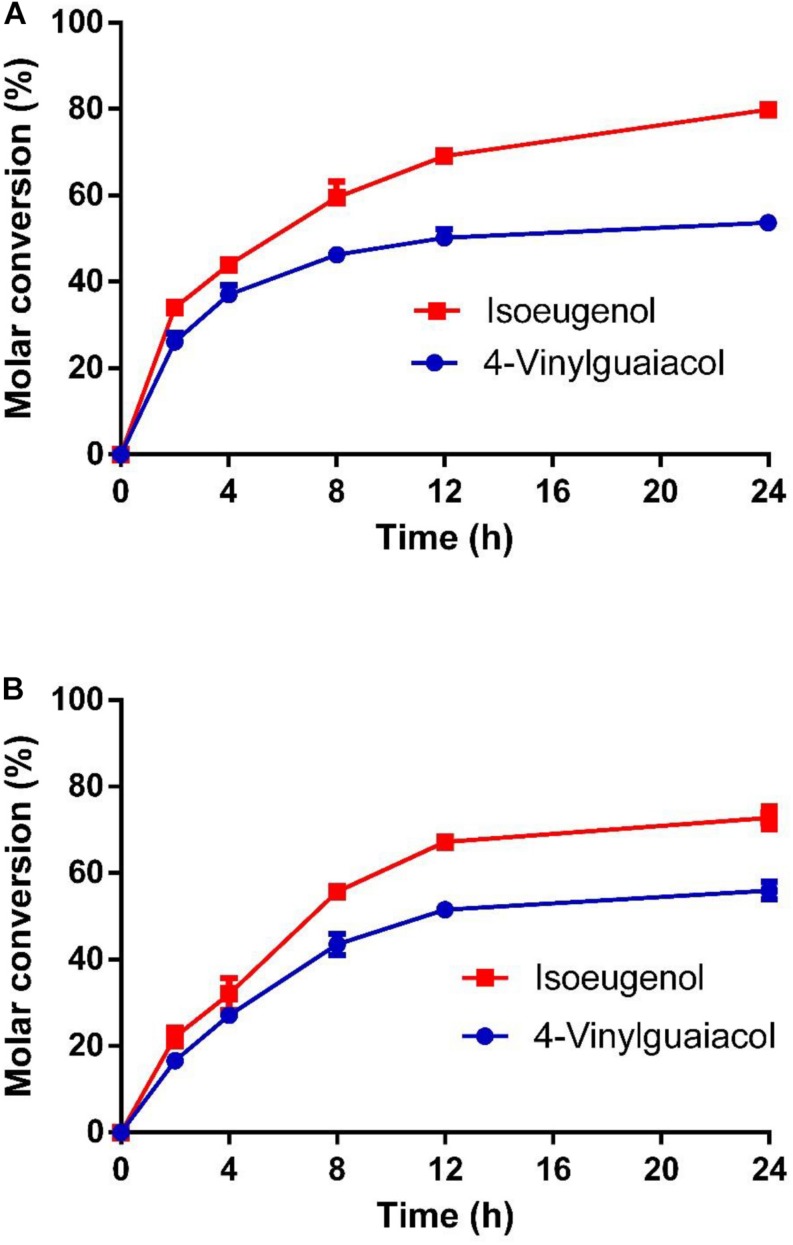
The conversion curve of whole cell catalysis of HsCCO **(A)** and RbCCO **(B)**. The whole cell catalysis was carried out under the optimal conditions without addition of organic solvent for 24 h. The samples were withdrawn at intervals and the molar conversion from 4-vinylguaiacol or isoeugenol to vanillin was calculated based on HPLC analysis. Values shown are means of triplicate means + standard error (SE).

As shown in Figure 7, the molar conversion yields of isoeugenol and 4-vinylguaiacol were 80 and 55% after HsCCO whole cell catalysis for 24 h. The molar conversion yields of isoeugenol and 4-vinylguaiacol were 75 and 58% after RbCCO whole cell catalysis for 24 h. It could be seen from the whole cell catalysis curve that the bioconversion is faster in the first 12 h but became slower in the later 24 h.

## Discussion

Five carotenoid oxygenase enzymes from different bacteria with different clusters in the phylogenetic tree were selected and their respective characteristics were compared in the study. Interestingly, enzymes with a similar cluster in the phylogenetic tree exhibited similar properties in terms of pH and solubility. All in the down cluster in the phylogenetic tree, the optimal pHs of AvCCO (pH 9.0), RbCCO (pH 9.0), RaCCO (pH 10.0), KsCCO (pH 9.5), and CsO2 (pH 10.5) were alkaline and most of the target proteins were in the insoluble fraction (Figures 2, 5, 6). While, in the up cluster in the phylogenetic tree, the optimal pHs of HsCCO (pH 7.0) and SeNCED (pH 8.0) from *Serratia* sp. ATCC 39006 (Tang et al., 2018) were close to neutral and the target proteins were highly soluble. By multiple alignment of amino acid sequences (Figure 1A), we found that some of the conserved residues of CCOs in the proteins with similar optimal pHs and expression solubility are identical (Red box, Figure 1A). It is speculated that these conserved amino acids may be the critical residues responsible for the pH optimum and solubility of CCOs.

Since KsCCO, AvCCO, and RaCCO were mainly expressed as insoluble proteins in *E. coli*, and furthermore, their catalytic activity was also relatively lower than HsCCO and RbCCO, so HsCCO and RbCCO was further investigated in this work. Carotenoid cleavage oxygenase contains four histidine’s that bind to Fe^2+^ (Kloer et al., 2005). The finding that the additional addition of Fe^2+^ in induction stage did not seem to improve the enzyme activity is different from other CCOs (Furuya et al., 2015; Tang et al., 2018). These observations could be explained that the trace Fe^2+^ in other chemicals used the culture media is sufficient to the formation of HsCCO and RbCCO activity center. The metal ions such as Co^2+^, Ni^2+^, and Zn^2+^ were reported to have negative effects on enzyme activity (Ryu et al., 2013; Tang et al., 2018). In this study, similar negative effects were observed for RbCCO activity. However, no inhibition effects were observed for HsCCO activity at presence of Co^2+^, Mg^2+^, and Zn^2+^, and Ca^2+^ at 5 mM even had an obviously stimulating effect on HsCCO activity, indicating the effects of metal ions were related to the sources of enzymes. Besides their difference in optimal pH and solubility of expressed proteins, HsCCO and RbCCO also differentiated in the temperature optimum and stability. HsCCO had a higher optimal temperature and thermal stability (25–35°C) than that of RbCCO.

Both enzymes showed the substrate preference for isoeugenol rather than 4-vinylguaiacol. The ratios of *V*_*max*_ of isoeugenol to 4-vinylguaiacol were 2.65 and 3.83 for HsCCO and RbCCO, respectively, which are significantly higher than previous reported SeNCED from *Serratia* sp. ATCC 39006 (Tang et al., 2018), but relatively lower than Cso2 (Furuya et al., 2014). So far, a few genes encoding isoeugenol monooxygenases have been identified originally from *P. putida* IE27 (PpIEM) (Yamada et al., 2007b), and *P. nitroreducens* jin1 (PnIEM) (Ryu et al., 2013), and a metagenomics DNA from soil (Zhao et al., 2018). The specific activity toward isoeugenol reached up to 5.53–9.20 U/mg, but the activity toward 4-vinylguaiacol was extremely low. In general, carotenoid cleavage oxygenase family enzymes catalyze the oxidative cleavage of conjugated C=C bonds of organic molecules. However, substrate preferences varied among these enzymes, the critical factors determining for the substrate specificity still needed to be elucidated in the future (Huang et al., 2009).

These differences in biochemical properties suggested that the reaction parameters needed to be optimized further before comparison of whole cell catalysis. Results revealed that the whole cell catalysis of HsCCO performed better at relatively higher temperature (25°C) but lower pH (pH 7.0) compared to RbCCO, consistence with their pH and temperature optima of activity. Higher shaking speed facilitated the catalytic efficiency of both enzymes due to better aeration, but a high concentration of substrate injured the catalytic efficiency due to the toxicity of substrate to enzymes (Furuya et al., 2015). The biphasic organic/aqueous system has many advantages over mono-aqueous system for biotransformation, especially if the substrate and product have low accessibility by the biocatalysts and toxic to biocatalysts (Eckstein et al., 2004; Wangrangsimagul et al., 2012). The catalysis efficiency of SeNCED was increased 1.7 times after adding 5% chloroform and 0.43 g L^–1^ vanillin was obtained after 72 h of reaction (Tang et al., 2018). In current work, the extra addition of an organic solvent somewhat displayed inhibition on the whole cell catalysis. It is worth noting that the whole cell catalysis mixture contained 5% dimethyl sulfoxide itself because of preparing the stock solution of substrate. It seems that 5% dimethyl sulfoxide might be enough for the catalysis. Addition of a higher concentration (over 10%) of organic solvent did not further enhance the production of vanillin as previous reports suggest (Furuya et al., 2015). In the final, current whole cell catalysis experiment, the whole cell molar conversion yields of HsCCO and RbCCO against isoeugenol were 80 and 75% and the molar conversion yields against 4-vinylguaiacol were 55 and 58%, respectively. The final vanillin yields of HsCCO and RbCCO were 1.22 and 1.14 g L^–1^ when isoeugenol was used as substrates; the final vanillin yields of HsCCO and RbCCO were 0.84 and 0.88 g L^–1^ when 4-vinylguaiacol was used as substrates, respectively. The whole cell catalytic yield was higher, and the catalytic time was shorter than SeNCED (Tang et al., 2018).

In summary, carotenoid oxygenase family protein sequences are widely distributed among various bacteria but most of their functions are still unresolved. In the current work, two promising CCOs were selected due to their superiority in biochemical properties compared to other putative CCOs from different bacterial strains. In comparison, HsCCO had a higher molar conversion yield for isoeugenol than RbCCO and was easier to be expressed as a soluble protein in *E. coli*, suggesting that HsCCO was a better choice for producing “nature vanillin” from isoeugenol and 4-vinylguaiacol than RbCCO. Our study provided a new insight in the functions of carotenoid oxygenase family protein sequences in bacteria. Further works need to be carried out to understand the determinants for their substrate specificity.

## Data Availability

The raw data supporting the conclusions of this manuscript will be made available by the authors, without undue reservation, to any qualified researcher.

## Author Contributions

ZH contributed to the study design, performed the bulk of the experimental work, data analysis, and interpretation, and drafted the manuscript. LL and SD contributed to the study design, data analysis, and interpretation, and revised the manuscript. All authors read and approved the final draft of the manuscript.

## Conflict of Interest Statement

The authors declare that the research was conducted in the absence of any commercial or financial relationships that could be construed as a potential conflict of interest.
