# Supplementary material for: Expression and Characterization of Carotenoid Cleavage Oxygenases From Herbaspirillum seropedicae and Rhodobacteraceae bacterium Capable of Biotransforming Isoeugenol and 4-Vinylguaiacol to Vanillin
Source: Front Microbiol. 2019 Aug 13;10:1869. doi: 10.3389/fmicb.2019.01869 (PMC6700365; doi:10.3389/fmicb.2019.01869)
Supplement: Supplementary file 1 [file Table_1.docx]

Supplementary Material


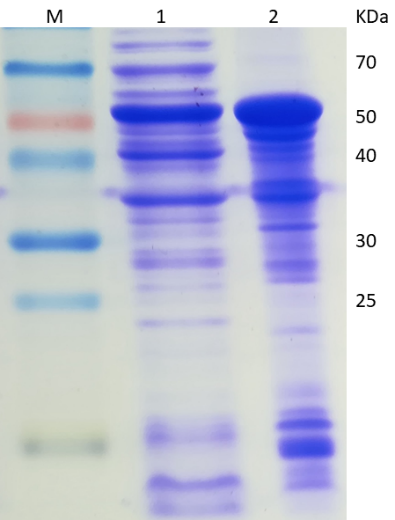


FIGURE S1 SDS-PAGE analysis of KsCCO. M: Protein marker. Lane 1: The supernatant fraction containing the target protein KsCCO. Lane 2: The precipitate fraction containing the target protein KsCCO.


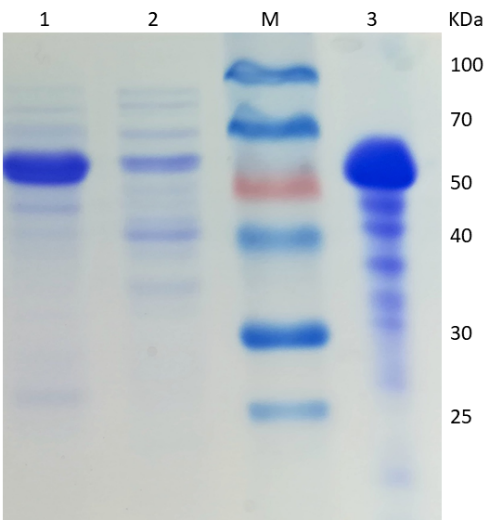


FIGURE S2 SDS-PAGE analysis of AvCCO. M: Protein marker. Lane 1: The puriﬁed AvCCO. Lane 2: The supernatant fraction containing the target protein AvCCO. Lane 3: The precipitate fraction containing the target protein AvCCO.


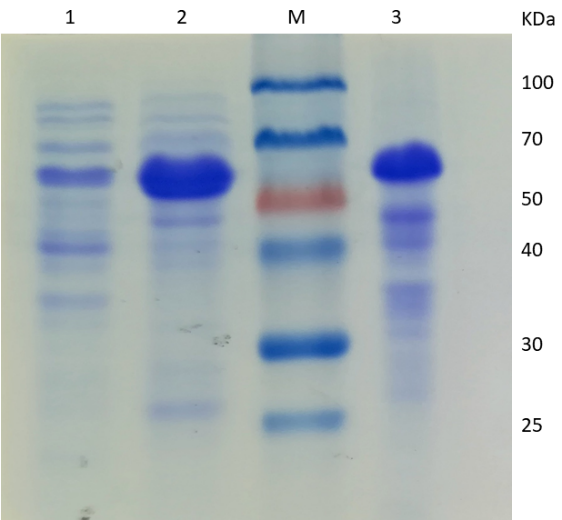


FIGURE S3 SDS-PAGE analysis of RaCCO. M: Protein marker. Lane 1: The supernatant fraction containing the target protein RaCCO. Lane 2: The puriﬁed RaCCO. Lane 3: The precipitate fraction containing the target protein RaCCO.


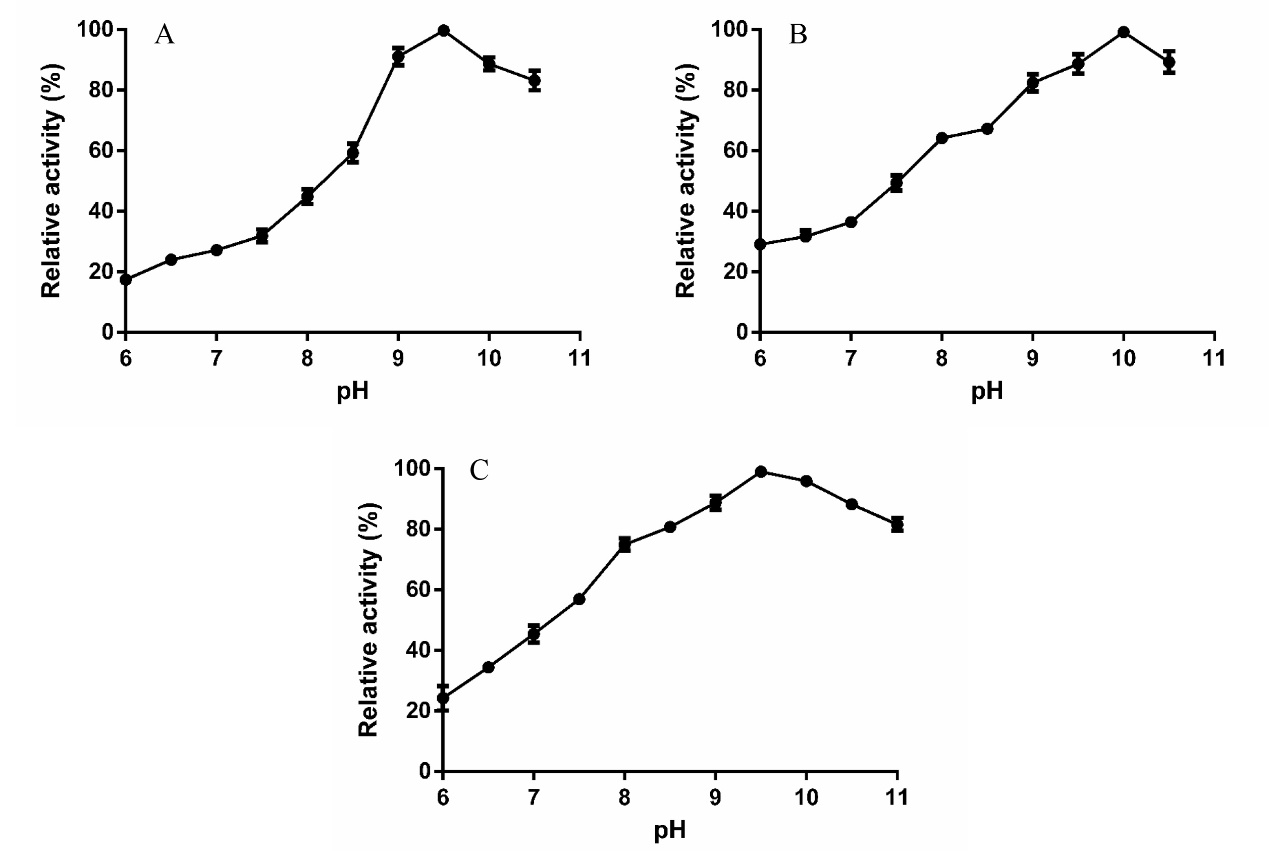


FIGURE S4. The optimal pHs of AvCCO (A), RaCCO (B) and KsCCO (C)


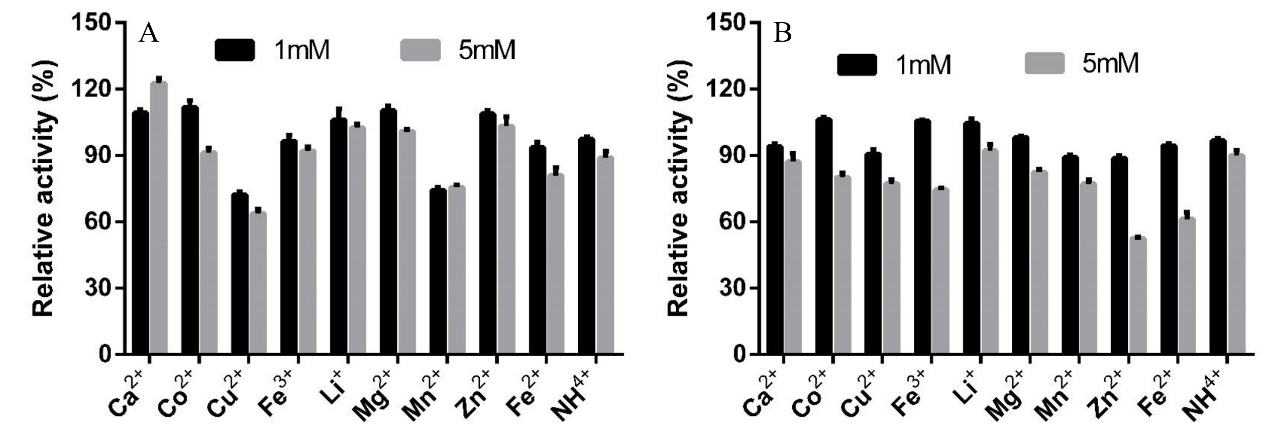
FIGURE S5. Effects of metal ions on HsCCO (A) and RbCCO (B) activity


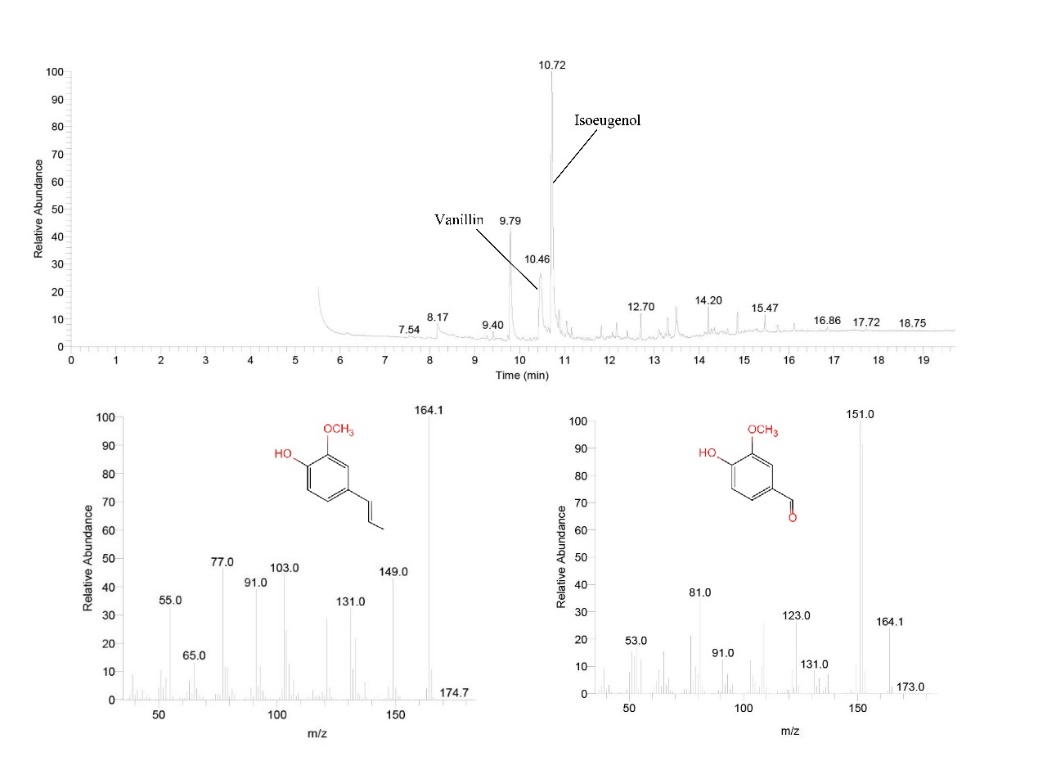


A_1_

A_2_

A_3_


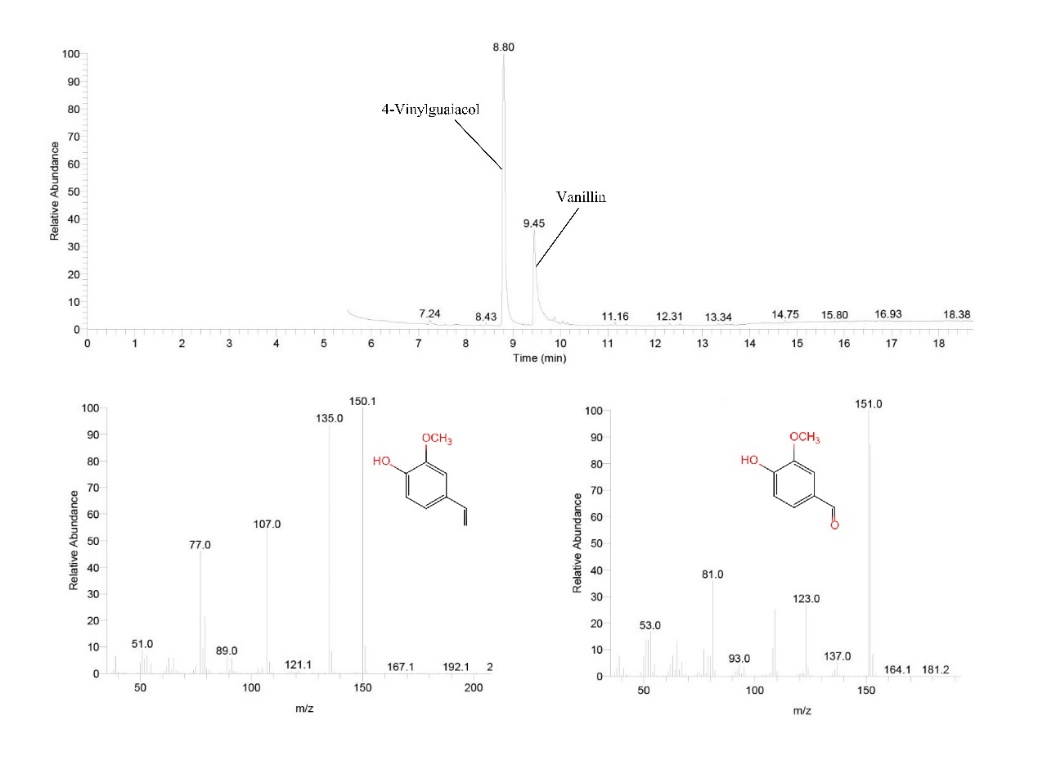


B_1_

B_2_

B_3_

FIGURE S6. GC-MS analysis of HsCCO catalysis product from isoeugenol (A) and 4-vinylguaiacol (B). (A1 and B1) Total ion current chromatogram of catalyzed sample; (A2 and B2) mass spectrogram of isoeugenol; (A3 and B3) mass spectrogram of the product vanillin. It should be noted that the column oven temperature programs were set to 60 ℃ for 2 min (4-vinylguaiacol as substrate) or 3 min (isoeugenol as substrate), then to 300 ℃ for 12 min with 20 ℃ min^−1^ due to different batch experiments, so the elution time of vanillin from 4-vinylguaiacol was 1 min faster than vanillin from isoeugenol.


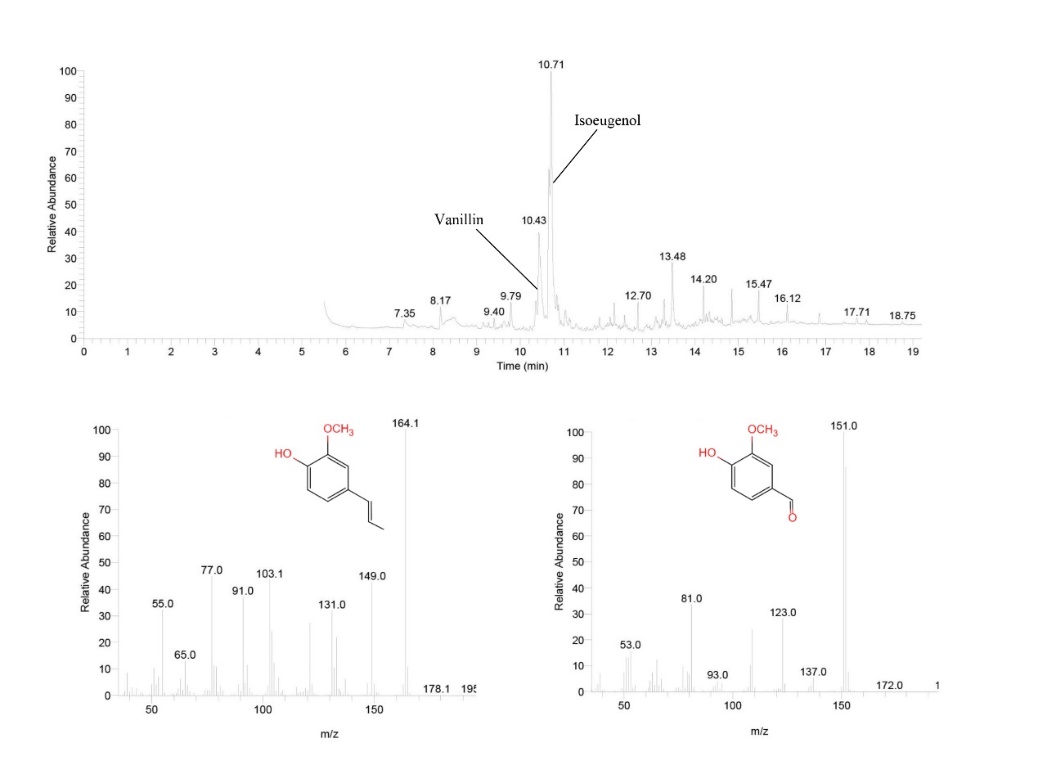


A_1_

A_2_

A_3_


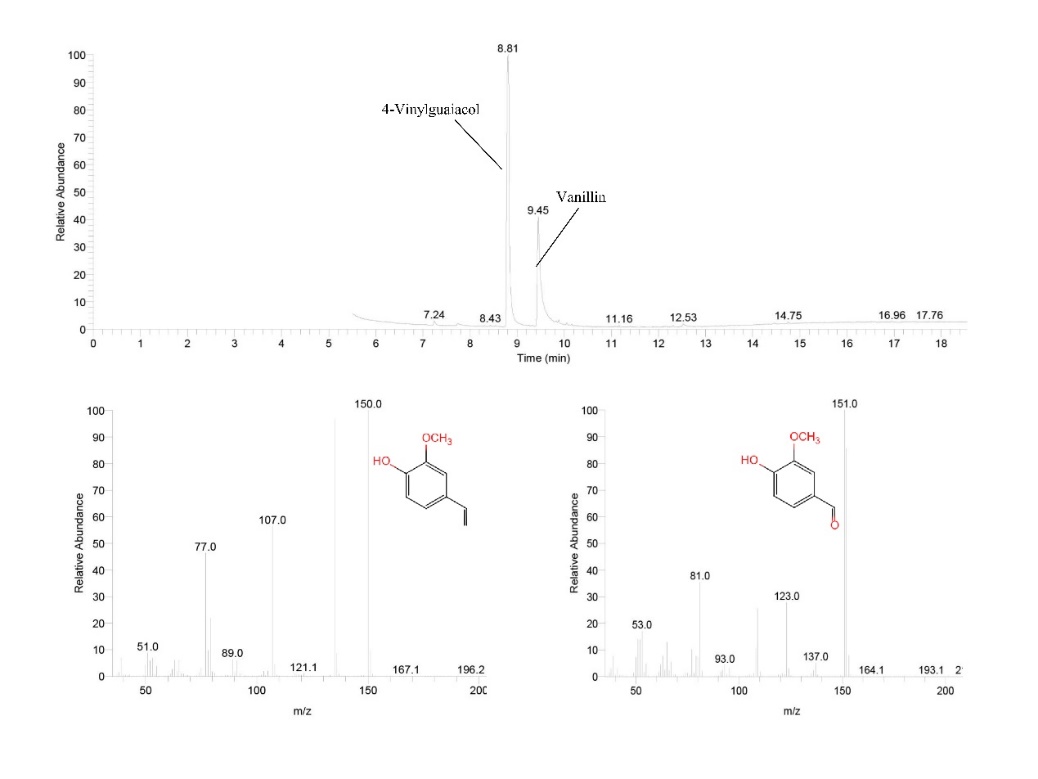


B_1_

B_3_

B_2_

FIGURE S7. GC-MS analysis of RbCCO catalysis product from isoeugenol (A) and 4-vinylguaiacol (B). (A_1_ and B_1_) Total ion current chromatogram of catalyzed samples; (A_2_ and B_2_) mass spectrogram of isoeugenol; (A_3_ and B_3_) mass spectrogram of the product vanillin. It should be noted that the column oven temperature programs were set to 50 ℃ for 2 min, then to 300 ℃ for 12 min with 20.8 ℃/min for 4-vinylguaiacol. While the temperature rising time from 50 ℃ to 300 ℃ was 13 min for isoeugenol due to different batch experiments, so the elution time of vanillin from 4-vinylguaiacol was 1 min faster than vanillin from isoeugenol..

TABLE S1 The enzyme names, organisms, and sequences used in the phylogenetic trees of carotenoid cleavage oxygenases of 22 amino acid sequences

| Enzyme names | Organisms | Sequences | Genbank accession no. |
| --- | --- | --- | --- |
| CsO2 | *Caulobacter segnis* | MTARFPNTREFTGALYRPSRFEGEVFDLEVDGQLPTDIDGTFFSVAPDAAFPPMREDDIFFNGDGAVSAFRFGGGHVDFQRRYVRTQRLEAQRAARRSLHGVYRNPSTNDPSVLGLNNSTANTNVLEHAGVLLAMKEDSLPYALDPLTLETKGLWNFGGQLTDAPFTAHPKIDPLTGDMIAFGYEARGDGSRDIVYYEFDEHGAKTREIWVQAPVSAMVHDFAVTERFVVFPIIPLSVDVERLRQGGRHFQWQPDLPQYFGVMRRDGDGGDLHWFTAPNGFQGHTLNAFDDGEKVYADMTSTNGNVFYFFPPADGFVPSPETLVSQLVRWTFDLSVKGGRLDMSPLTPFPAEFPRIDDRVALRPHRHGWMMAMDPTKPYAEDRVGPRPFQFFNQLAHLNIATGKIQTWFADEASCFQEPVFVPRTGSSREGDGYLLSLVNRLDERTTDMVVLDALRLGEGPVATVKLPLRMRMALHGNWSRAVSPSSIKAV | WP_013078876.1 |
| RbCCO | *Rhodobacteraceae bacterium* GWE1_64_9 | MTRPFPKTPEFSGSLYRPARFEGSVFDLEVEGRVPEDIHGTFFQVAPDPQFPPMRGNDIFFNGDGAVHAFRFANGAVDFQRRYVMTDRLKAQRAERESLFGLYRNGYTNDPRADGVHMGTANTNVVQHNGRLLALKEDSPPYALDPVTLETHGLFDFDGQLTSPTFTAHPKFDPETGAMLCFGYEAKGEATPDIVYYEIDSQGRKQREIWLRGPYAAMIHDFAVTRNYIIFPLMPLTPDLDRIRKGGNHFEWQPGLEQVFGVLPRDGDGTDVRWFTAPNGFQGHTLNAFDDGRGRIFVDMPVTDGNIFYFFPQSDGHVPPPETLSSRMMRWTFDMNNPGNALSMAPITDFACEFPKCDPRYSGHEYRHGFVIGMDMTRPFDAERLGAPPFQFFNLLSHVDVATRRVKSWFADAQTCFQEPVFVPRSANAPEGEGWVIALANRLVDQTTDLVILDAQHMDEGPVAVVHLPVRLRMSLHGSWVPAAA | OHC44943.1 |
| KsCCO | *Kaistia soli* | MTVPFPAEPEFSGALYEPARFEGDVFDLIVEGNLPATIDGVFYQVAPDPQFPPMLGQDIFFNGDGCIHSFTFQNGHVDFRRRYVMTERLLAQRAERRSLHGVYRNTFTNDPSVAGISNSTANTNVVRHGGLLLALKEDSPPYALDPVTLETRGLWDFNGQLTSATFTAHPKIDPETGDLLCFGYEAKGEATPDIVYYEIDRTGRKKRETWLRGPYAAMIHDFAVTENYVIFPLMPLTADLDRIKQGGRHFQWQPGLEQLFGVLPRNGEAKDVRWFTAPNGFQGHTLNAFDDGKGRVYVDMPVTSGNIFYFFPQSDGRVPPPETLKSQLTRWTFDMGRPGNNLESTPLTQFPCEFPKSDDRYMGRPYRHGFVIAFDPAKPYDGERLGPPPFQFFNMLAHVDVATQKTKTWFSDDQTCFQEPIFVPKSENAAEGEGYVMALANRLRDRVTDLVVLDAQHLDEGPIATVKLPFRLRMSLHGSWAPAEH | WP_073057496.1 |
| AvCCO | *Agrobacterium vitis* | MTVPFPNKPEFTGSLYKPARFEGQVYDLEVEGKVPEEIDGTFFQVAPDPQYPPMLGEDIFFNGDGAVSAFRFKNGHVDFQRRYVMTERLKAQRDARASLHGIYRNPFTNDPSVKDISNSTANTNVVVHNGKLLALKEDSPPYALDPITLETIGLYDFDGQLTSATFTAHPKFDPETGDLLCFGYEAKGEATPDIVYYEIDKHGRMKREVWITAPYAAMIHDFAVTEHFVIFPLMPLTADLERMKQGGKHFQWQPGLDQLFGILRRDGDGRDVRWFKAPNGFQGHTLNAFDDGGRIFVDMPVTSGNIFYFFPQSDGTVPPPETLSSQMMRWTFDMRSNGNNIEVKPLTSFACEFPRSDDRYCGRQYRHGFVIAMDPTKPFDEARIGPRPFQFFNQLAHLDIATGKTQLWFADDQSCFQEPIFVPRRPDAPEGDGYVIGLVNRLAERATDLLVLDAQHLSDGPIATIKLPMRLRMSLHGNWVPGDQLKAV | WP_012654221.1 |
| RaCCO | *Rhodococcus aetherivorans* | MTTEFPDVPALSGWEEPLRFEGEVRDLEIIGEVPAEIDGAFFRVAPDPFYPNRTGTDIFFNGDGNVGAFRIKDGNVDFRQRYVQTDRFLAERKAGRSLFGAYRNPFTDDPSVAGLSRSTANTNVVVHNGTLWALKEDSLPIAMDPETLETKGFSDFGGKMRSRTFTAHPKIDPHTGEMICFGYAAKGEALPDIAYYVISASGEVTHEAWFEAPYASMIHDMAITDNYVVFPVMPLGSSIERMKRGGMHFQWEPEWPVYYGVIPRRGNGEDVRWFRAPNAFPGHTLNAHEKDGRILLDISLTLGNVFPWFPPASGDVTDPATLPTRFARVALDLRSTTKEAETFELLDIIAEFPHIDDRFVGTDYRHGFFAGMDLSVPLRLDRVRNKPFNLVFNSLVHGDAHTGKVKYWTPGETDTVQEPVFVPRSANAAEGEGFVMLLVNRLAEGRSDLVILDSTDIEGGPLAVAKLPMRMKFGLHGNWADASTFTGVTTDVQPRRV | WP_029541323.1 |
| SeNCED | *Serratia* sp. ATCC 39006 | MSLKFPETPEFTGLYKPSRVETQVFDLEIEGEVPPQIKGTFFQVSPDSYYPPMQGKDIFFNGDGLVSAFKFENGHVSLRRRYVQTDRLKAQWKERRSLNGIYRNIYTNDPLAAENNTTANTTVLFHAGVLLAMKEDALPYALNPNTLETLGVWDFNGQITSATFTAHPKIDPDNGDLLCFAYEAKGDGTSDIAYFEIDASGKLKKEIWFKGPYAAMIHDFAVTAHHVVFPLIPLTADVDRMKAGGQHFEWQPDLPQLFGVLPRDGSSEDVLWFHGPKDGFQGHTLNAYEEDGLLRVDMPVTNGNVFYFFPQADGSVPLPETLSSQLMRWTFNLNGPGESEAGQQTLQPQPLTSFPCEFPRCDERFTGKPYEHGFVLAFDPALPFDETLGERPFQFFNQLAHVNVRTGETETWYPGNAQCFQEPIFVPRSPDAPEGDGYVIALLNHLHGNDTELVVLDSLKMATGPVARIKVPFRLRMSLHGNWTPAEALKGTQSGR | WP_021015152.1 |
| HsCCO | *Herbaspirillum seropedicae* | MSMQYPATPEFQGMYRPSRVEASVQALEVVGQLPAQLQGCFYQVAPDPAYPPMLGQDIFFNGDGMVSAFRFGQGQVSLTRRYVQTERLKAQRQAGRSLNGIYRNIYTNAPEAASDNTTANTTVIKHAGVLLALKEDSLPYAMDPVTLETRGLWDFHGQVKSATFTAHPKICPQSGSMLCFAYEAKGDGTPDIAYYEIDAAGKLIRETWFQAPYAAMIHDFAVTENYVIFPIIPLTVDVERMKRGGQHFQWQPDLPQLFGILPRAGDAADVRWFYGPADGFQGHTLNAYELGRKLILDMPVTSGNVFYFFPQADGFVPSPETLKSGLARWTFDLDADNDQVMPEPLTQFPCEFPRCDERYSGRPYQHGFMLAFDPTLPFDAAALGAPPFQFFNQLAHVNVRTGETRTWYAGDAHCFQEPVFVPRSADAPEGDGFLLSLLNDLRSGATELVILDTHDLSAGPVARVQIPLRMRMSLHGNWSSD | WP_069373448.1 |
|  | *Acinetobacter baumannii* | MSFTFPNTSEFTGLYEPCRIEADITDLVIEGDIPSAIKGTFYQVAPDPQYPPMLGNDIFFNGDGVVTAIELGEGRVSMKRRYVQTPRLVAQKQAHRSLNGVYRNIYTNDPLAAKNNTTANTTVIEHNGVLLAMKEDALPWALDLKTLETIGEWDFNGQINSATFTAHPKIEPKTGNLLCFAYEAKGDGTPDIAYYEISATGELLKEIWFQAPYAAMIHDFAVTENYVIFPIIPLTVDIERMKKGGQHFQWQPDLEQLFGILPRSGQAEDVQWFYGPKNGFQGHTLNSFEKNGKIYVDMPVTSGNVFYFFPPAEAPVHSEQITSALMRWEFDLQATDHHVKPQPITNKQYPCEFPRCDERFNGLEYSYGFLLAFDPDLAFDHENLGEYPFQFFNQLARVNVQTGITETWYPGDKYCFQEPIFIPRSNDASEGDGWVASIMNDLLEEKSELVILDTQNWEKGPIARVKIPFRLRMSLHGNWSPQEK | WP_031996858.1 |
|  | *Marinomonas profundimaris* | MSFTFPNTPEFTGLYKPSRIEAEVKDLEIEGELPKEINGTFYQVAPDPQYPPMLGTDMFFNGDGMVSAFQFDTGSVSLTRRYVKTDRLMAQRREQRSLNGIYRNQYTNDPLAADNNTTANTTVIEHNGVLLALKEDALPWAMDLNTLETLGEWDFDKQIKSATFTAHPKIDVDNGALLCFAYEAKGDATPDIAYFEISKDGKLTKEIWFQAPYAAMIHDFAVTDNYVIFPVIPLTVDVERMKAGGKHFQWQPDLPQLFGVLPRNGSAEDVRWFYGPSNGFQGHTLNSYEEEGKLIVDMPVTSGNVFYFFPQADGHVPSPQSLKSALMRWVFDLNSEESQVEPVMITENPFPCEFPRCDARYSGKKYSWGFLLAFDPSLPFDFASLGEPPFQFFNQLARININTGKSETWYPGDAHCFQEPIFIPRSATADEGDGWVVSVMNNLREESSELVVMDTASWSDGPIAKVKVPFRLRMSLHGNWSGA | WP_024022327.1 |
|  | *Pectobacterium carotovorum* | MSLQFPNIPEFTGLYQPSRVEVSVVDLEVEGTLPEAIKGTFYQVAPDPHYPPMLGKDIFFNGDGIVSAFRFENGRVSLQRRYVETERLKAQRREHRSLNGTYRNVYTNDPLAADNNTTANTTVIEHNGVLLAMKEDALPWAMDLDTLETLGEWDFHGQITSATFTAHPKVDPDTGALLCFAYEAKGEATPDIAYFEISAEGKLVREIWFQAPYAAMIHDFAVTQNYVVFPIIPLTADLDRMKSGGQHFQWQPDLPQLFGIVPRDGSADDVRWFYGPANGFQGHTLNAYEEGSKIFVDMPVTNGNVFYFFPEEGGYVPPPEALKAALMRWTFDLNADDTTVTPQLLTRTPFPCEFPRCDERYSGKPYHYGFTLAYDPQLPFDTATLGAPPFQFFNQLARINVVTGESEMWYPGDAQCFQEPIFIPRHAQAAEGDGWVVCVMNDLRKRTSDLIILDTAQWTQGPVARVKVPFRLRMSLHGNWSGK | WP_039354738.1 |
|  | *Paraburkholderia tropica* | MSSQFPSTPEFVGLYQPSRVEADVTDLEVEGTLPDCISGVFYQVAPDPQYPPMLGNDIFFNGDGAVCAFRFDHGKVSLKRRYVQTDRLRAQRRERRSLNGVYRNVFTNDPQAAQNNSTANTTVLKHNGVLLAMKEDNLPYAMDPDTLETFGVWDFDGQVRSATFTAHPKVDPETGDLLCFAYEAKGDGTPDIAYFEIGAFGELKREVWFKAPYAAMIHDFAITEHHVVFPVIPLTVDVERMKNGGPHFQWQPDLPQLFGVLPRLGSADDIRWYYGPANGFQGHTLNSFEEGSRIHVDMPVTGGNVFYFFPQEDGSVPPPETLKASLMRWTFDLDATINKVEPKALMDFPCEFPRCDERYTGRPYRHGFVLKFDPGLPFDGENLGAPPFQFFNEIAHVDVVSGDTEAWFAGDAECLQEPIFVPRSPDAPEGDGFVISVLNHLRSQTSELVVLDGMKMSSGPVARIKIPFRLRMSLHGSWSPL | WP_074986642.1 |
|  | *Xanthomonas hyacinthi* DSM 19077 | MSMLFPTTPEFTGLYKPSRVEADVVDLEIEGEVPAELAGVFFQVSPDSHYPPMLGKDIFFNGDGLVSAFRFDRGRVSLRRRYVQTDRLLAQRREGRSLTGVYRNVYTNDPLAAANNTTANTTVLFHGGVLLAMKEDALPYALDPHTLQTRGVWDFGGQITSATFTAHPKLDPDNGDLLCFAYEAKGDGTPDIAYFEIDAAGRLKKEVWFKAPYAAMIHDFAVTEKHVVFPLIPLTVDLERMRQGGQHFQWQPDLPQLFGVLPRDGGAEDIRWFTGPKDGFQGHTLNAYEEHGRLHLDMPVTSGNVFYFFPQADGSVPSPETLRSQLMRWTFDLDAKGGEIEPVALTEFMCEFPRCDERYSGRPYRHGFVLAYDPTLPFDASLGAPPFQFFNQLAHVDVVAGTSEAWFPGDAQCFQEPIFVPRAEDAPEGDGYVIALLNHLHGENTELVVLDSLRMADGPVARIKLPLRMRMSLHGNWTPAKALAALHD | KLD77535.1 |
|  | *Pseudomonas fulva* | MTALFPETPEFSGALYRPSRVEADVMDLEIEGELPAAIRGAFYQVSPDHQYPPLLGNDIFFNGDGMVSAFFFADGKVSLRRRYVQTDRLLAQRHEGRSLNGIYRNVYSNDELAAANNTTANTTVLKYGDVLLAMKEDALPYAMDPQTLETLGVYDWNGQIKSATFTAHPKIDPVSGNLLAFSYEAKGDGTPDMAYFEISPDGKLLKEIWFQAPYAAMVHDCAITPNYIVFPFIPLTVDVERMKRGGQHFQWQPDLPQLFAVLPRHGTASDVRWFKGPANGFQGHTLNAFEQDGQIHVDMPVTDGNVFYFFPQQDGFVPNPETLSSTLQRWTFDLSAKSDLVEPRPLTDYRCEFPRVDERYTGLPYEHGFMLSFDPTRPYREENGPMPFQFFNLLTHFNLRTGVSDSWFPGESGCFQEPIFVPRSADAPEGDGYVIALLNNLFDFSSELVVLDSRDMATGPVARIRIPFRMRMSLHGSWSAAE | WP_013791154.1 |
|  | *Pseudomonas fuscovaginae* | MTAQFPKIPEFSGSLYYPSRAEVDVFDLEIEGSLPADIKGAFYQVSPDPQYPPMLGEDIFFNGDGLVSAFFFENGRVSLRRRYVKTDRLMAQRREGRSLNGIYRNVHTNDSKAAKNNTTANTTVLMYNGVLLAMKEDALPYAMDPITLETLGVHDFDAQIKAQTFTAHPKVDPVTGNLLAFSYEAKGDGSPDLAYFELAPDGTLQHEIWFQAPYAAMVHDFAVTEHHVVFPLIPLTVDVERMQRGGQHFEWQPDLPQLFAIVPRRGSSSDVHWFKGPANGFQGHTLNAFEENGLIHVDMPVTSGNIFYFFPQADGFVPSPETLNSQMMRWSFDLSADSDEVEPFALSEFRCEFPRCDDRFVGRPYEHGFVIAFDPEKPYNAANGPMPFQFFNLLTHMNVRTGESESWFAGDSECFQEPIFVPRSADAPEGDGYVMALLNHITVHSTELVVLDSLNMAAGPIARIKLPLRMRMSLHGSWATTQ | WP_010446183.1 |
|  | *Pseudomonas poae* | MSTRFPQTPEFSGALYSPSRVEAEVLDLEVEGTLPASILGVFYQVAPDPQYPPMLGSDMFFNGDGIVSGFYFADGKVSLRRRYVKTDRLLAQRREGRSLNGVYRNAYTNDPLAAKNNTTANTSVIPHNGRLLALKEDALPWAMDLQTLETLGEWDFAGQIKSATFTAHPKIDPVTGNLLAFSYEAKGDGTPDMAYFELSPDGNLLKEIWFQAPYAAMVHDFAVTEHYVVFPLIPLTVDVERMKNGGPHFQWQPDLPQLFAVLPRNGNAQAIRWFKGPKDSFQGHTLNAFDQDGKVYVDMPVTGGNVFYFFPQADGAVPVPEDLPSSLMRWTFDLNDARDDVEPQPLTDYMCEFPRCDERYLGRPYEHGFVLAFDPTLPYNPANGPMPFQFFNQLAHLNLKTGSTDAWFPGDNSCFQEPIFIPRSADAEEADGYVVALLNVLDQARSELVILDSRDMASGPIARIKVPLRMRMSLHGCWAQG | WP_098479824.1 |
|  | *Pseudomonas lurida* | MNTPFPNTPEFSGALYAPSRVEGDVYDLEIEGALPEAIRGAFYQVSPDPQYPPMLGSDIFFNGDALVSGFYFADGKVSLRRRYVLTDRLVAQRREGRSLNGVYRNVYTNDPLAAKNNTTANTSVIPHNNVLLALKEDAMPWALDPQTLETLGEWNFDGQIDAATFTAHPKLDPVTGNLLAFSYEAKGDGTPDMAYFEISPEGKLLHEIWFQAPYAAMVHDFAVTERYVVFPLIPLTVDVERMKKGGQHFQWQPELPQLFAVVPRHGHARDVRWFKGPKDGFQGHTLNAFDEDGKVYVDMPVTGGNIFYFFPQADGFVPPPETLAASLMRWTFDLNSAQDELQPQALTDYPCEFPRCDDRYIGRKYEHGFLLAFDPERPYNPSNGPMPFQFFNLLVHLNLTTGVTDAWFPGDSGCFQEPIFIPRSAQAAEGDGYVVCLLNLIAEGRSELVVLDATDMAAGPLARIKIPFRMRMSLHGCWAPR | WP_098466954.1 |
|  | *Pseudomonas brassicacearum* | MSIPFPQTPEFSGALYKPSRIEAEVFDLEIEGVLPASIHGTFYQVAPDPQYPPMLGTDIFFNGDGMVSGFHFANGKVSLRRRYVQTDRLLAQRREGRSLNGVYRNAFTNDSLAAKNNTTANTSVIPHNGVLLALKEDALPWAMDLETLETLGEWTFDGQIKSATFTAHPKLDPATGNLLAFSYEAKGDGTPDLVYFELSPDGKLLHEIWFQAPYAAMVHDFAATERYVVFPLIPLTVDVERMKNGGPHFQWQPDLPQLFAVVPRNGRAQDVRWFKGPMDGFQGHTLNAFDEDGKVYVDMPVTGGNIFYFFPQADGHVPPPETLAACLMRWTFDLNSGRDEVEPQPLTDYPCEFPRCDDRYIGRQYAHGFLLAFDPERPYNPANGPIPFQFFNLLVHLNLKTGLSDAWFPGDSGCFQEPIFIPRSADAEEADGYVVALLNLIAEERSELVVLDSRDMASGPIARIRIPFRMRMSLHGCWAPGS | WP_025212951.1 |
|  | *Pseudomonas fluorescens* | MSILFPQTPEFSGVLYTPSRVEAEVFDLEIEGTLPASICGTFYQVAPDPQYPPMLGNDIFFNGDGVVSRFNFANGKVSMRRRYVKTDRLLAQRREGRSLNGVYRNVYTNDPLAAKNNTTANTTVIPHNGVLLALKEDALPWALDLETLETLGEWTFDGQIKAATFTAHPKLDPVTGNLLACSYEAKGDGTPDLAYFELSPDGKLLHEIWFQAPYAAMVHDFAVTERYVVFPLIPLTVDVERMKNGGPHFQWQPDLPQLFAIVPRYGSAQDVRWFKGPKDGFQGHTLNAFDEDGKVYVDMPVTGGNIFYFFPQADGYVPPPETLAASLMRWTFDLNGAQEDVQPQPLTKYPCEFPRCDDRYIGRKYQHGFLLAFDPERPYNPANGPIPFQFFNLLAHLDLQTGLTDAWFPGDSGCFQEPIFIPRSADAEEADGYVVALLNLIAEGRNELVVLDTRDMASGPIARIRIPFRMRMSLHGCWAPSD | WP_003173119.1 |
|  | *Pseudomonas kilonensis* | MSIPFPQTPEFSGALYTPSRVEAEVFDLEIEGILPASICGTFYQVAPDPQYPPMLGNDIFFNGDGVVSRFNFANGKVSMRRRYVKTDRLLAQRREGRSLNGVYRNVYTNDPLAAKNNTTANTTVIPHNGVLLALKEDALPWALDLETLETLGEWTFDGQIKAATFTAHPKLDPVTGNLLACSYEAKGDGTPDLAYFEISPDGKLLHEIWFQAPYAAMVHDFAVTERYVVFPLIPLTVDVERMKNGGPHFQWQPDLPQLFAIVPRNGCGQDVRWFKGPKDGFQGHTLNAFDEDGKVYVDMPVTGGNIFYFFPQADGYVPPPETLAASLMRWTFDLNGAQEDVQPQPLTEYPCEFPRCDDRYIGRKYQHGFLLAFDPERPYNPANGPIPFQFFNLLAHLDLQTGRTDAWFPGDSGCFQEPIFIPRSADAEEADGYVVALLNLIAEGRSELVVLDTRDMASGPIARIRIPFRMRMSLHGCWAPND | WP_046064917.1 |
|  | *Massilia* sp. JS1662 | MSHCFPDSREFSGPLYRPSRVEADVFDLEVEGTVPPEIEGVFYQVSPDPQFPPMLGDDIFFNGDGMVSAFRFEKGQVSLRRRYVQTDRLLAQRRAGRSLTGVYRNVYTNDPAAAANNTTANTGVLEHGGVVLALKEDGLPYALDRDSLETIGRWNFAGQVKSATFTAHPKIDPATGDLLAFGYEAKGDGTRDIAYFEIGKDGALKKEVWFEAPYAAMIHDFAVTENFVIFPVIPLTVDVERMKAGGRHFEWQPDLPQLFGVMPRNGGANDVRWFKGPVNSFQGHVLNAFDRDGKAYMDMPVVGGNVFYFFPQADGFVPPPESLAPNLVRWTFDLDSASDQVVPEPLANLICEFPRCDDRYVGRPYRHGFMLAFDPTLPYDGRRLGPPPFQFFNQLAHFDIQTGRSETWFAGDKESFQEPIFVPRSRTAPEGDGYVIALLNHLGSESTSLVVLDSRNMPAGPIARIRIPFRMRMSLHGSWSPRAF | WP_036234943.1 |
|  | *Sphingobium yanoikuyae* | MTSCFPDDPIYRGFDAPGRVEANVFDLEVEGRVPPELDGTFFRVAPDPQWPPMLGHDIFFNGDGMVCAFRFKDGRVDFTSRYAQTDKFVAERQARKALYGAYRNPYTDDPSVAGSIRSTANTNVIVHHGLLLALKEDSPPVAMRPDTLETIGNYRFGDKMMSETFTAHPKVDPVSGELIAFGYSAKGVATSDLAYYVIDRHGEVVHEAWFTAPRAASIHDFAVTENYVVFPVGSHEIETERLKAGKPAFVWRPDVEQIYGVLPRRGNAEDMRWFTVPTNGFQGHTINAWDDGHKVYVDMPMLNDNAFWFYPDENGHAPHPSTLKQTMTRWIFDLSSNSVTPQMDIIPAPMGEFPHIDERYATRPYRHAFLAVIDPTAPYDFQRCGPPSVNAFLNGLAHVDMTTGASRRWLPGPTSTVQEPVFAPRSPESPEGDGYVIALVNRLDEMRSDLVVLDAQHIDEGPVATIRLPLRLRNGLHGNWVPSSAMRSPLPA | ARQ83690.1 |
|  | *Penicillium subrubescens* | MEAKVEHAFPDRPQFQGFMKPCRMEGEVQALEVLGDIPKEIDGTFYRVMPDPQLPPFVQDDPWFNGDGNIAAFTIKDGTVTFRQRYVRTEKFIREREAKRALLGKYRNKYTDAVEFKIRTTANTNVVYFNGQLLALKEDAPPYALDPITLETKGLYDFEGQLPSLTFTAHPKFDPKTGEMICFGYEAKGDGTPDVCYYSVSPDGKFTEVVWLVAPVAAMIHDFAVTENWVLFPIIPQVCDIERMKQGGEHWQWSPETPVYLGVLPRRGAKPTDVKWFEYKNSFPGHTVNAYEDTNGHITLDLGLSSKNVFFWWPDAAGNAPEPSSILSQIVRFTLDPNSKGEALHLPSPKILQKDNTEFYRIDDRFATQPHRHCFMDLMDPTLGTDFQTIAPRLGGGYPLYNSLAHLDIETGETEIYFPGRTHMVQEPVFIPRKGSRVEGDGFLLALVNNYESMGSELHLVDTRDFTKVKAVILLPIRLRQGLHGNWVDAVDLKVSI | OKP00514.1 |

TABLE S2: Specific activities of five purified CCOs using isoeugenol as a substrate

|  | AvCCO | RaCCO | HsCCO | RbCCO | KsCCO |
| --- | --- | --- | --- | --- | --- |
| Specific activity (nmol min^-1^ mg^-1^) | 44.92 | 23.45 | 69.51 | 68.45 | 50.23 |
